# Compendium of TCDD-mediated transcriptomic response datasets in mammalian model systems

**DOI:** 10.1186/s12864-016-3446-z

**Published:** 2017-01-13

**Authors:** Stephenie D. Prokopec, Kathleen E. Houlahan, Ren X. Sun, John D. Watson, Cindy Q. Yao, Jamie Lee, Christine P’ng, Renee Pang, Alexander H. Wu, Lauren C. Chong, Ashley B. Smith, Nicholas J. Harding, Ivy D. Moffat, Jere Lindén, Sanna Lensu, Allan B. Okey, Raimo Pohjanvirta, Paul C. Boutros

**Affiliations:** 1 Informatics and Bio-computing Program, Ontario Institute for Cancer Research, 661 University Avenue, Suite 510, Toronto, ON M5G 0A3 Canada; 2 Department of Pharmacology & Toxicology, University of Toronto, Toronto, Canada; 3 Department of Veterinary Biosciences, University of Helsinki, Helsinki, Finland; 4 Department of Biology of Physical Activity, University of Jyväskylä, Jyväskylä, Finland; 5 Department of Environmental Health, National Institute for Health and Welfare, Kuopio, Finland; 6 Laboratory of Toxicology, National Institute for Health and Welfare, Kuopio, Finland; 7 Department of Food Hygiene and Environmental Health, University of Helsinki, Helsinki, Finland; 8 Department of Medical Biophysics, University of Toronto, Toronto, Canada

**Keywords:** TCDD, AHR, Microarray datasets, R

## Abstract

**Background:**

2,3,7,8-tetrachlorodibenzo-*p*-dioxin (TCDD) is the most potent congener of the dioxin class of environmental contaminants. Exposure to TCDD causes a wide range of toxic outcomes, ranging from chloracne to acute lethality. The severity of toxicity is highly dependent on the aryl hydrocarbon receptor (AHR). Binding of TCDD to the AHR leads to changes in transcription of numerous genes. Studies evaluating the transcriptional changes brought on by TCDD may provide valuable insight into the role of the AHR in human health and disease. We therefore compiled a collection of transcriptomic datasets that can be used to aid the scientific community in better understanding the transcriptional effects of ligand-activated AHR.

**Results:**

Specifically, we have created a datasets package – TCDD.Transcriptomics – for the R statistical environment, consisting of 63 unique experiments comprising 377 samples, including various combinations of 3 species (human derived cell lines, mouse and rat), 4 tissue types (liver, kidney, white adipose tissue and hypothalamus) and a wide range of TCDD exposure times and doses. These datasets have been fully standardized using consistent preprocessing and annotation packages (available as of September 14, 2015). To demonstrate the utility of this R package, a subset of “AHR-core” genes were evaluated across the included datasets. *Ahrr*, *Nqo1* and members of the *Cyp* family were significantly induced following exposure to TCDD across the studies as expected while *Aldh3a1* was induced specifically in rat liver. *Inmt* was altered only in liver tissue and primarily by rat-AHR.

**Conclusions:**

Analysis of the “AHR-core” genes demonstrates a continued need for studies surrounding the impact of AHR-activity on the transcriptome; genes believed to be consistently regulated by ligand-activated AHR show surprisingly little overlap across species and tissues. Until now, a comprehensive assessment of the transcriptome across these studies was challenging due to differences in array platforms, processing methods and annotation versions. We believe that this package, which is freely available for download (http://labs.oicr.on.ca/boutros-lab/tcdd-transcriptomics) will prove to be a highly beneficial resource to the scientific community evaluating the effects of TCDD exposure as well as the variety of functions of the AHR.

**Electronic supplementary material:**

The online version of this article (doi:10.1186/s12864-016-3446-z) contains supplementary material, which is available to authorized users.

## Background

The aryl hydrocarbon receptor (AHR) is an evolutionarily conserved transcription factor [1] activated by small molecule binding. Prior to ligand-activation, the AHR resides in the cytoplasm bound to chaperone proteins, including heat shock protein 90 (HSP90) and the AHR-interacting protein (AIP) [2, 3]. Ligand-activation of this receptor leads to translocation into the nucleus, dissociation of chaperones and dimerization with the AHR nuclear translocator (ARNT) [4]. The AHR: ARNT complex is able to bind DNA at recognized motifs known as aryl hydrocarbon response elements (AHREs) whereby transcription of the associated genes is regulated [5]. Modulation of AHR activity has been linked to various diseases, including numerous in vitro studies of breast [6–8], endometrial [9], kidney [10], lung [11, 12] and prostate [13] cancers and inflammatory skin [14] and bowel [15, 16] diseases.

Activation of the AHR can occur by both endogenous molecules, such as tryptophan metabolites [17], and exogenous molecules, such as polycyclic aromatic hydrocarbons. 2,3,7,8-tetrachlorodibenzo-*p*-dioxin (TCDD) is the most potent congener of chlorinated dioxins, a large class of environmental contaminants produced as a by-product of various industrial processes [18]. Many of the toxic effects of TCDD exposure, including chloracne, immunosuppression, hepatotoxicity and cancer [19], are mediated by the AHR. Strong evidence for this relationship comes from studies of AHR-knockout mice [20–22], mice containing AHR-inactivating mutations [23–25] and conditional ARNT-null mice [26], all of which are unresponsive, or show reduced response, to TCDD. Differences in the toxic outcomes of TCDD occur across species and have been linked to polymorphisms in the AHR. A particularly TCDD-resistant strain of mice, DBA/2 J, presents with an Ala375Val mutation within the *AHR* gene; this leads to reduced affinity of the receptor for TCDD [27–29]. As another example, two strains of rat, Long-Evans (L-E) and Han/Wistar (H/W) show dramatic differences in TCDD susceptibility. These differences are primarily due to a point mutation that results in partial deletion of the transactivation domain of the *AHR* in the TCDD-resistant H/W rat [30]. Furthermore, inbred lines (Line A/B/C) derived from L-E x H/W crosses demonstrate intermediate susceptibility to TCDD depending on AHR genotype [31]. In humans, the AHR gene most closely resembles that of the DBA/2 J mouse [29]. However, a number of polymorphisms have been identified within both the ligand-binding and transactivation domains of the human AHR [32] but the overall functional consequences of these polymorphisms are not yet clear. In addition to the differences in TCDD-response phenotypes among different mammals, TCDD exposure results in tissue-specific responses. In rats, TCDD tends to accumulate in liver, spleen, adipose tissue and pancreas [33]. The broadest spectrum of transcriptional responses in rodents is detected in liver [34]. Further compounding the issue, there is a sex-dependent element to the transcriptomic alterations evoked by TCDD [35–38].

Despite considerable study into the transcriptomic changes mediated by the TCDD: AHR complex that lead to the observed toxic outcomes, the specific genes and pathways responsible for these outcomes remain unknown. As such, a global resource describing transcriptomic changes following activation of the AHR across a wide variety of tissues and species would prove very useful to the scientific community. Therefore, we have generated such a resource consisting of transcriptomic data from numerous studies in our laboratory and others (a total of 377 samples) and we introduce the freely-available TCDD.Transcriptomics package for the R statistical environment.

## Implementation

### Experimental methods

The experimental design, animal handling and sample preparation for individual experiments are described elsewhere [34, 37–43]. Data from human Multipotent Adipose-Derived Stem (hMADS) cells (differentiated and undifferentiated; TCDD treated and control) were downloaded from NCBI’s GEO repository (GSE32026) [42], as were data for primary human and female Sprague–Dawley (SD) rat hepatocyte cell lines (GSE14555) [44]. Array data for DBA/2 J mouse liver were generated in our laboratories as follows: briefly, adult male DBA/2 J mice were bred in the colonies of the National Public Health Institute, Division of Environmental Health, Kuopio, Finland. Study plans were approved by the Finnish National Animal Experiment Board (Eläinkoelautakunta, ELLA; permit code: ESLH-2008-07223/Ym-23). Animals were housed singly in Makrolon cages with aspen chip bedding (Tapvei Oy, Kaavi, Finland) and provided with Altromin 1314 pellet feed (Altromin Spezialfutter GmbH & Co. KG, Lage, Germany) and water available *ad libitum*. The housing environment was maintained at 21 ± 1 °C, with a relative humidity of 50 ± 10% and a 12 h light cycle. TCDD (5 or 500 μg/kg dissolved in corn oil) or corn oil vehicle alone were administered by oral gavage (10 mL/kg). Animals were euthanized by carbon dioxide, followed immediately by cardiac exsanguination 19 h following treatment. Livers were excised and frozen in liquid nitrogen. Tissue samples were shipped on dry ice to the analytic laboratory and stored at −80 °C.

Similarly, adult male transgenic “AHR-ratonized” mice, ages 12–23 weeks, were bred as above. Mice were housed singly in suspended, wire-mesh stainless-steel or Makrolon cages, with the housing environment maintained as described above. Animals were provided with R36 pellet feed (Lactamin, Stockholm, Sweden) and tap water available *ad libitum*. Animals (n = 83) were divided into 5 groups per *Ahr* isoform (INS/DEL/rWT) and TCDD (125, 250, 500, or 1000 μg/kg dissolved in corn oil) or corn oil vehicle alone were administered by oral gavage (10 mL/kg). Animals were euthanized by cervical dislocation 4 days following exposure. Tissue was collected and stored as above.

Animal handling and reporting comply with ARRIVE guidelines [45]. RNA isolation and microarray hybridization were performed as described elsewhere [37, 38, 46]. Remaining data were generated and deposited in the GEO repository as described in the original publications [34, 37–41, 43].

### Computational methods

#### Human cell lines

For hMADS cells, raw GenePix image data (.gpr files) were loaded into the R statistical environment (v3.2.1) using the limma (v3.24.13) package. Efforts were made to emulate the processing procedures conducted by the original authors: images were first cleaned by filtering out bad spots (flagged as “bad”, “not found” or “absent” or known as −100, −50 and −75 respectively) by assigning those spots a weight of zero. Normalization was performed within arrays by applying global LOESS. Agilent Feature IDs were annotated with EntrezGene IDs and gene names using an annotation table obtained from NCBI's GEO repository for the array type (GPL4133). Linear modelling was conducted to identify genes with statistically significant differential mRNA abundances between TCDD-treated and reference samples. An empirical Bayes method was applied following model fitting to reduce standard error and moderated t-tests were used to assess statistical significance [47]. All *p*-values were adjusted for multiple testing using a 5% false discovery rate (FDR) [48]. Genes with multiple mapped Agilent Feature IDs were trimmed by keeping the feature ID with the lowest *p*-value.

For primary human hepatocyte cell line data, CEL files were downloaded from GEO (GSE14555) and loaded into the R statistical environment (v3.3.1) using the affy package (v1.48.0) of the BioConductor library [49]. Due to limited sample size, data for both sexes and all dose points were processed together. Data were normalized using the RMA algorithm [50] and probe annotations were obtained using the custom CDF [51] hgu133ahsentrezgcdf (v19.0.0) and database hgu133ahsentrezg_db (v19.0.0) packages. Probes were filtered using a background intensity threshold established by evaluating chromosome Y associated probes in female samples. Linear modelling was performed using the limma (v3.28.21) package, with contrasts fit to identify differences between treatment and control groups for each treatment dose. An empirical Bayes moderation of the standard error [47] was applied, and model-based t-tests were used to assess significance, accompanied by FDR correction for multiple testing [48].

#### Mouse data

Raw CEL files for livers from male and female C57BL/6 mice treated with TCDD or corn oil along a time-course (GSE61037) were loaded in the R statistical environment (v3.2.1) using the affy package (v1.46.1)as described above. Data for both sexes and all time points were preprocessed together and normalized using the RMA algorithm [50]. Probe annotations were obtained using the custom CDF [51] mogene11stmmentrezgcdf (v19.0.0) and database mogene11stmmentrezg_db (v19.0.0) packages. Chromosome Y probe filtering was performed as described above. Linear modelling was performed separately for each time point with both male and female samples using the limma (v3.24.13) package. An empirical Bayes moderation of the standard error [47] was applied, and model-based t-tests were used to assess significance, accompanied by FDR correction for multiple testing [48].

Data from the corresponding dose–response study were processed similarly with the following exceptions: male and female samples were preprocessed and modelled separately to avoid masking sex-specific effects at low dose treatments. As such, no additional probe filtering was performed. Similarly, transgenic “AHR-ratonized” mouse data were preprocessed and modelled independently for each AHR genotype with no additional probe filtering. Dose–response data from DBA/2 J mouse liver were processed and modelled as a single dataset using the above methods.

#### Rat data

For each experiment, raw CEL files were loaded in the R statistical environment (v3.2.1) using the affy package (v1.46.1) of the BioConductor library [49]. Rat adipose data (GSE18301) were preprocessed as a whole, including both strains and time points, using the RMA algorithm [50] with the custom rat2302rnentrezgcdf (v19.0.0) package [51]. Probes were further annotated with gene symbols and named using the rat2302rnentrezg_db (v19.0.0) package. Linear modelling was performed on all samples using the limma (v3.24.13) package, with contrasts fit to specify individual comparisons for both strains. Experiments at the day-1 time-point evaluated TCDD relative to corn oil, whereas the 4-day experiment compared TCDD with feed-restricted, corn oil treated controls. As above, an empirical Bayes moderation of the standard error [47] was applied, and model-based t-tests were used to assess significance, accompanied by FDR correction for multiple testing [48]. Identical processing was performed for data generated from rat hypothalamus at 23 h (GSE61039) and rat liver at 4 and 10 day time points (GSE13513). Data from rat liver, 3 and 19 h time points (GSE10083), were processed together as described above using the custom rae230arnentrezgcdf (v19.0.0) package [51].

CEL files for the primary rat hepatocyte cell line dataset were downloaded from GEO (GSE14555) and loaded into the R statistical environment (v3.3.1) using the affy package (v1.48.0) of the BioConductor library [49]. Data were normalized using the RMA algorithm [50] and probe annotations were obtained using the custom CDF [51] rgu34arnentrezgcdf (v19.0.0) and database rgu34arnentrezg_db (v19.0.0) packages. As this specifc array type does not include probes for genes located on chromosome Y, no additional filtering was performed. Linear modelling was performed using the limma (v3.28.21) package, with contrasts fit to identify differences between treatment and control groups for each treatment dose. An empirical Bayes moderation of the standard error [47] was applied, and model-based t-tests were used to assess significance, accompanied by FDR correction for multiple testing [48].

#### Interspecies comparisons

All interspecies comparisons may be facilitated using homologene IDs provided by NCBI. A function is provided in the TCDD.Transcriptomics package to load filtered HomoloGene data (build 68) for comparison of mouse, rat and human transcriptomic responses to TCDD.

#### Package access

The TCDD.Transcriptomics package is available for download from http://labs.oicr.on.ca/boutros-lab/tcdd-transcriptomics, along with instructions for direct download and installation within the R statistical environment. TCDD.Transcriptomics is designed for use with the R statistical environment (≥ v2.10.1) and is dependent on the BoutrosLab.plotting.general (BPG) package (≥ v3.10.2) (P’ng et al., submitted) for production of plotting covariates and easy integration with data visualizations. The BPG package can be found here: http://labs.oicr.on.ca/boutros-lab/software/bpg.

## Results and Discussion

In recent years, activation of the AHR has become the focus of myriad studies across a wide range of fields. In particular, significant effort has gone into elucidating the mechanism by which TCDD activation of the AHR leads to a diversity of toxic outcomes. As the ligand-activated AHR is a transcription factor, the transcriptome has been the primary focus of these studies. As such, we have collated a number of transcriptomic datasets arising from various TCDD exposure studies in rats, mice and human cell lines (Table 1, Additional file 1) into a datasets package for the R statistical environment. Specifically, microarray data from 12 separate studies covering 63 unique experimental conditions were collected. Data were generated using two microarray platforms and seven unique array types: Agilent’s Whole Human Genome Microarray and Affymetrix’s Human Genome U133A Array, Rat Expression Array (230A), Rat Genome U34 and 230 2.0 Arrays, Mouse Genome 430 2.0 Array and Mouse Gene 1.1 ST Array. Data from Affymetrix arrays were processed using the RMA algorithm with the most recent probe to EntrezGeneID map (as described in Materials and Methods), analyzed using linear modelling, FDR-corrected for multiple testing and results format-standardized.

**Table 1:** Summary of TCDD Datasets

Species	Tissue	Strain/AHR Genotype	Sex	Number of Samples	Dose(s) (μg/kg)	Time(s) (hours)	GEO Accession
Human	adipose-derived stem cells	hWT	N/A	8	25nM	48	GSE32026
	primary hepatocyte cell line	hWT	male and female	3	0.00001, 0.001, 0.01, 0.1, 1, 10, 316 (nM)	48	GSE14555
Mouse	kidney	C57BL/6	male	12	0, 1000	19	GSE15857
		AHR-KO	male	6			
	liver	C57BL/6	male	62	0, 125, 250, 500, 1000	6, 19, 24, 72, 96, 144	GSE15858 GSE61037 GSE61038
			female	47		6, 24, 72, 96, 144	GSE61037 GSE61038
		DBA/2	male	12	0, 5, 500	19	NA
		AHR-KO	male	6	0, 1000	19	GSE15858
		rWT (L-E)	male	17	0, 125, 250, 500, 1000	96	GSE72270
		DEL (H/W)	male	21			
		INS (H/W)	male	22			
Rat	adipose	L-E	male	20	0, 100	24, 96	GSE18301
		H/W	male	8		24	
	hypothalamus	L-E	male	7		23	GSE61039
		H/W	male	8			
	liver	L-E	male	39		3, 19, 96, 240	GSE10083 GSE13513
		H/W	male	30			
		Ln-A (H/W)	male	8		19	GSE10083
		Ln-C (L-E)	male	8			
	primary hepatocyte cell line	rWT (SD)	female	14	0.00001, 0.001, 0.01, 0.1, 1, 10, 316 (nM)	48	GSE14555

A total of 63 datasets are included in this package consisting of 377 samples across 3 species, 4 tissue types and a wide range of AHR genotypes, TCDD exposure times and doses

Each dataset is labelled with a highly descriptive title, indicating the species, strain, tissue type, length of exposure (hours), TCDD dose (μg/kg) and sex (male if unlabelled) from which the data were generated. Furthermore, each dataset contains three parts: the sample information, preprocessed data and analysis results. First, the sample information file outlines the type and treatment conditions for each sample in the experiment. The second file contains the expression data and consists of the normalized array data for all samples listed in the sample information file. Finally, the results file outlines the magnitude and significance of change as determined by linear modelling for each gene available on the array. In this file, all genes are annotated with EntrezGene IDs, gene symbol and full gene name.

**Fig. 1:**
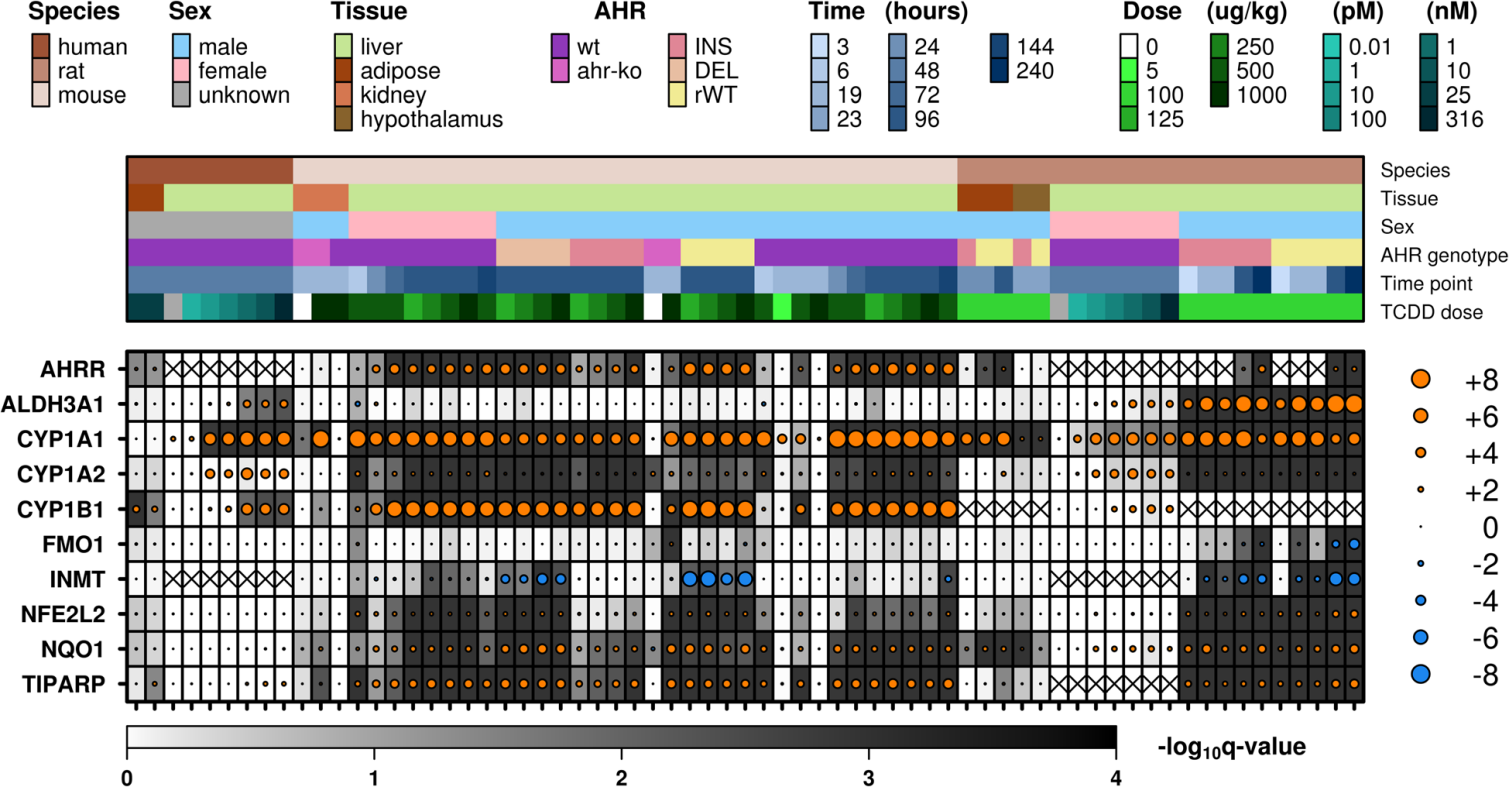
“AHR-core” genes. Transcriptional response (treatment relative to control) of a subset of 10 “AHR-core” genes across the available datasets demonstrates unexpected variability. Dot size indicates magnitude of change in mRNA abundance; colour represents direction of change; background shading demonstrates significance of change. Boxes containing an ‘X’ indicate that data for the given gene was not available for the indicated dataset

To provide an example of the usefulness of this R package, a set of widely recognized “AHR-core” genes, genes with mRNA transcription known to be regulated by the AHR transcription factor in a wide range of species and tissues [52–57], were visualized across the available datasets (Fig. 1). Data was extracted according to Homologene ID in order to ensure the suitability of comparisons across species. Interestingly, while most of the selected genes show some degree of TCDD-mediated change in transcript levels across the datasets, some genes demonstrate a species and/or tissue specific effect. In particular, ALDH3A1 appears to be induced only in rat liver and primary human hepatocytes. Similarly, INMT is repressed in livers of rats and TCDD-sensitive “AHR-ratonized” mice. The variability in response to the “AHR-core” genes highlights the need for further study into the effects of TCDD on various organisms.

While the TCDD.Transcriptomics package provides access to a wide range of datasets, it is by no means fully comprehensive. The GEO repository contains a number of additional datasets relating to array-based mRNA abundance studies of TCDD-mediated transcriptional regulation, however these typically pertain to gestational exposure in mice and rats, human cancer-derived cell lines and zebra fish – all of which are beyond the current scope of this package but may be incorporated at a later date.

## Conclusion

Here we have produced a standardized compendium of TCDD-mediated transcriptional changes for use in the R statistical environment. This package, titled TCDD.Transcripomics, contains data from 63 experiments including 377 samples, incorporating data from 3 species, 4 tissue types, both sexes and a wide range of AHR genotypes, TCDD exposure times and doses, along with an up-to-date HomoloGene dataset for interspecies comparison, and is freely available for download (http://labs.oicr.on.ca/boutros-lab/tcdd-transcriptomics). This datasets package will provide a significant resource for the scientific community encompassing a variety of fields of study, from toxicological studies concerning TCDD and dioxins to studies of the AHR and its role in normal physiology and human disease.

### Availability and requirements

Project name: TCDD Transcriptomics

Project home page: http://labs.oicr.on.ca/boutros-lab/tcdd-transcriptomics

Operating system(s): tested on Ubuntu and Debian

Programming language: R

Other requirements: depends on BPG (http://labs.oicr.on.ca/boutros-lab/software/bpg)

Licence: GPL-2

## Additional file

Additional file 1: Overview of Included TCDD Datasets. (XLSX 16 kb)

## Abbreviations

AHR: Aryl hydrocarbon receptor
H/W: Han/Wistar (*Kuopio*)
hMADS: Human Multipotent Adipose-Derived Stem
L-E: Long-Evans (*Turku/AB*)
SD: Sprague Dawley
TCDD: 2,3,7,8-Tetrachlorodibenzo-*p*-dioxin
